# The Spread of *Mycoplasma pneumoniae* Is Polyclonal in Both an Endemic Setting in France and in an Epidemic Setting in Israel

**DOI:** 10.1371/journal.pone.0038585

**Published:** 2012-06-06

**Authors:** Sabine Pereyre, Alain Charron, Carlos Hidalgo-Grass, Arabella Touati, Allon E. Moses, Ran Nir-Paz, Cécile Bébéar

**Affiliations:** 1 Université Bordeaux, USC Infections Humaines à Mycoplasmes et Chlamydiae, Bordeaux, France; 2 INRA, USC Infections Humaines à Mycoplasmes et Chlamydiae, Bordeaux, France; 3 Centre Hospitalier Universitaire de Bordeaux, Laboratoire de Bactériologie, Bordeaux, France; 4 Hadassah-Hebrew University Medical Center, Jerusalem, Israel; Miami University, United States of America

## Abstract

*Mycoplasma pneumoniae* infections occur both endemically and epidemically, and macrolide resistance has been spreading for 10 years worldwide. A substantial increased incidence of *M. pneumoniae* infections has been reported in several countries since 2010. Whether this increased incidence is attributed to different or to the same *M. pneumoniae* genotype is unknown. We have developed a multilocus variable-number tandem-repeat (VNTR) analysis (MLVA) for the molecular typing of *M. pneumoniae* isolates. In this study, the MLVA typing method was modified and validated to be applicable directly to respiratory tract specimens without culture. This method was applied to 34 *M. pneumoniae*-positive specimens received at the Bordeaux Hospital, France, between 2007 and 2010 in an endemic setting, and to 63 *M. pneumoniae*-positive specimens collected during an epidemic surge of *M. pneumoniae* infections in 2010 in Jerusalem, Israel. The *M. pneumoniae* endemic spread was shown to be polyclonal in France, with 15 MLVA types identified. Strikingly, the Israeli epidemic surge was also a multi-clonal phenomenon, with 18 circulating MLVA types. The macrolide resistance-associated substitution, A2058G, was found in 22% of the Israeli patients. Macrolide-resistant *M. pneumoniae* belonged to four MLVA types, the MLVA type Z being the most frequent one. An association between the MLVA type Z and macrolide resistance might exist since macrolide resistance was present or generated during the course of illness in all patients infected with this MLVA type. In conclusion, the discriminatory power of the MLVA showed that the spread of *M. pneumoniae* strains in France in an endemic setting was polyclonal as well as the surge of *M. pneumoniae* infections in Israel in 2010.

## Introduction

*Mycoplasma pneumoniae* infections occur both endemically and epidemically worldwide, especially in children and young adults [1]. A substantial increased incidence was reported since 2010 in several countries of Northern Europe [2], [3], [4] and Israel [5], [6]. Whether this increased incidence is attributed to different or to the same *M. pneumoniae* genotype is unknown. To date, the typing methods of *M. pneumoniae* do not allow recognition of a clonal distribution of a strain. Indeed, until recently, the most common typing methods of *M. pneumoniae* were based on the analysis of the gene encoding the P1 protein. Several typing methods based on this gene were developed including PCR-restriction fragment length polymorphism (RFLP) [7], [8], amplification and gene sequencing [9], real-time PCR with high-resolution melt analysis [10] and pyrosequencing [11]. However, isolates were poorly differentiated due to the separation of isolates into only two types and a few variants related to each type [12], [13], [14], [15].

We developed a multi-locus variable-number tandem-repeat (VNTR) analysis (MLVA) for the molecular typing of *M. pneumoniae* isolates. This method is based on one-step multiplex PCR amplifications using labelled primers and can differentiate over 26 distinct VNTR types [16]. A correlation was found with the P1 gene-based typing methods, but MLVA has a higher discriminatory power. Later, a culture-independent method was developed [17], but it remained labour-intensive and time consuming due to the use of five independent nested PCR reactions and electrophoresis. MLVA typing was recently applied during an increase of *M. pneumoniae* infections in England and Wales [2]. However, MLVA profiles were only obtained for 17 out of 22 samples, which was not sufficient to assess the diversity of *M. pneumoniae* types circulating during this epidemic surge.

Additionally, macrolide-resistant *M. pneumoniae* have been spreading for 10 years worldwide, with prevalences ranging from below 10% in Europe, approximately 30% in Israel [5] and up to 90% in Asia [18]. The possibility of an association between macrolide resistance and a specific *M. pneumoniae* clone has never been assessed due to the lack of a discriminatory genotyping method.

To determine whether the *M. pneumoniae* spread is clonal or polyclonal, we improved the MLVA typing protocol to allow for typing of *M. pneumoniae* directly in respiratory tract specimens without culture. This method was applied to 34 *M. pneumoniae*-positive specimens received at the Bordeaux Hospital, France, over the course of a 3-year endemic period and to 63 *M. pneumoniae*-positive specimens collected in Jerusalem, Israel, during 2010, at which time an epidemic surge of *M. pneumoniae* infections was observed. Resistance to macrolides was also assessed for a possible association between the MLVA type and macrolide resistance.

## Materials and Methods

### Ethics Statement

The present project is in compliance with the Helsinki Declaration (Ethical Principles for Medical Research Involving Human Subjects).

The French study was conducted in accordance with the guidelines of the “Direction de la Recherche Clinique et de l’Innovation", the research board of Bordeaux University hospital, Bordeaux, France. All patient data were anonymously reported, with no possibility of connecting the isolates and specimens to individual patients. Using the written “livret d’accueil" of the Bordeaux University Hospital, patients are explicitly informed at the admission to hospital that their samples could be used for research purposes and that they can oppose to this use. As specimens used in this study are part of routine patient management without any additional sampling, and since patients provided no objection for their samples to be used, the article L1211-2 of the French code of Public Health states that this study did not need to be examined by the ethical committee “Comité de Protection des Personnes" and that patient’s informed consent was not required.

The study at the Hadassah-Hebrew University medical center was approved by the Hadassah-Hebrew University medical center institutional review board without the need for informed consent, as part of the retrospective study on the characteristics of patients infected with *M. pneumoniae*. All samples were analysed anonymously for both resistance and typing.

### Clinical Specimens and M. Pneumoniae Isolates

To compare the MLVA typing results between clinical isolates and their original corresponding clinical specimens, 18 *M. pneumoniae* isolates previously typed by the MLVA method [16] were randomly selected. The 18 corresponding respiratory tract specimens, stored at the Department of Bacteriology in the University Hospital of Bordeaux, France, were thawed and subjected to direct MLVA typing. The results were compared.

Over three years (October 1st 2007–September 30th 2010), all the respiratory tract specimens detected positive for M. pneumoniae by the in-house real-time PCR [19] at the Department of Bacteriology in the University Hospital of Bordeaux, France, were retrospectively recorded. Available remnants of 34 specimens or DNA extracts stored at −80°C were thawed. The M. pneumoniae isolates that could be grown from the clinical specimens [20] were also used for MLVA typing. DNA was extracted using the MagNA Pure LC DNA isolation kit I (Roche Diagnostics, Frpance) according to the manufacturer’s instructions.

Additionally, 63 throat-swab specimens positive for *M. pneumoniae* by in-house real-time PCR [5] collected from 55 patients were used. These specimens were collected at the Hadassah-Hebrew University Medical Centre, Jerusalem, Israel during an epidemic surge of *M. pneumoniae* respiratory infections between January 1^st^ 2010 and December 31^st^ 2010. The throat-swab specimens collected between January and August 2010 have previously been described [5]. DNA was extracted by boiling, as previously described [5].

### Detection of Macrolide-resistance-associated Mutations

French isolates and respiratory tract specimens were analysed using the real-time PCR and melting curve analysis method [21]. The presence of macrolide resistance-associated mutations was confirmed by amplification and sequencing of the domain V of the 23 S rRNA gene [21]. Throat specimens from Israel were analysed for mutations using a real-time PCR and high-resolution melt analysis method directly from boiled extracts [5], [22], and the presence of mutations was confirmed by sequencing.

### MLVA Typing

MLVA typing on *M. pneumoniae* isolates was performed using two multiplex PCRs with fluorescently labelled primers targeting five VNTR loci, followed by capillary electrophoresis [16]. The method was adapted to be used directly on DNA extracts from clinical specimens. Modifications concerning the sample amount and the number of PCR cycles were required to amplify *M. pneumoniae* DNA from clinical specimens. Briefly, the reaction mixtures M1 and M2 [16] both corresponding to multiplex PCRs were prepared in a final volume of 25 µl, with 5 µl of template used instead of 1 µl for bacterial isolates. Both multiplex mixtures were run using the same cycling conditions of 95°C for 15 min, followed by 40 cycles of 95°C for 1 min, 60°C for 1 min and 72°C for 1 min instead of 25 cycles for clinical isolates, before a final extension of 72°C for 10 min. Electrophoresis and GeneScan analysis were performed as previously described [16]. When an inconsistency in the MLVA type was found between the *M. pneumoniae* isolate and the clinical specimen, the relevant VNTR locus was analysed from both the isolate and the corresponding specimen using the nested PCR method [17].

## Results

### Comparison of MLVA Typing Results between Respiratory Tract Specimens and Corresponding *M. pneumoniae* Isolates

Our MLVA typing protocol was modified to allow for direct typing on respiratory tract sample DNA extracts. To validate the modifications, we initially tested 18 *M. pneumoniae*-positive respiratory tract specimens from which 18 *M. pneumoniae* isolates had been grown and had previously been typed by our MLVA method [16] (Table 1). Direct typing was successful in 13 out of 18 clinical specimens (72%). All specimens that could not be typed had low levels of *M. pneumoniae* DNA. This was deduced because the cycle thresholds exceeded 35 for the real-time PCR targeting the P1 adhesin gene that was used for primary detection of the pathogen. The MLVA types were identical between clinical isolates and their original clinical specimens.

**Table 1:** Comparison of MLVA typing results between 18 *M. pneumoniae* isolates and their corresponding respiratory tract specimens.

M. pneumoniae isolates							Corresponding clinical specimens						
M. pneumoniae isolate designation	Mpn 1	Mpn 13	Mpn 14	Mpn 15	Mpn 16	MLVA type^a^	Cycle threshold of the corresponding clinical specimen^b^	Mpn 1	Mpn 13	Mpn 14	Mpn 15	Mpn 16	MLVA type
B4074	4	4	5	7	2	P	31.6	4	4	5	7	2	P
B4098	6	3	5	6	2	V	33.6	6	3	5	6	2	V
B4104	5	3	5	6	2	S	35.2	5	nd^c^	nd	6	2	nd
B4112	4	3	5	6	2	M	36.2	4	3	5	6	2	M
B4209	5	4	5	7	2	U	36.6	nd	nd	nd	7	nd	nd
B4229	5	4	5	7	2	U	33.9	5	4	5	7	2	U
B4254	3	3	6	6	2	H	21.4	3	3	6	6	2	H
B4381	4	3	6	6	2	O	34.6	4	3	6	6	2	O
B4391	3	3	6	7	2	I	38.0	3	3	6	6	2	I
B4466	5	3	5	6	2	S	27.9	5	3	5	6	2	S
B4470	4	3	6	6	2	O	21.8	4	3	6	6	2	O
B4550	3	4	5	7	2	J	39.9	nd	nd	nd	nd	nd	nd
B4543	4	3	6	6	2	O	22.7	4	3	6	6	2	O
B4567	4	3	6	6	2	O	37.1	nd	nd	nd	nd	nd	nd
B4578	2	4	5	7	2	E	28.9	2	4	5	7	2	E
B4594	5	3	6	6	2	T	37.9	5	nd	nd	nd	2	nd
B4608	5	3	6	6	2	T	23.6	5	3	6	6	2	T
B4628	2	3	5	6	2	B	21.9	2	3	5	6	2	B

a MLVA types of *M. pneumoniae* isolates were previously determined by Degrange *et* *al.* [16].

b Cycle threshold from the in-house real-time PCR targeting the *M. pneumoniae* adhesin P1 [19] used for the primary detection of the pathogen.

c nd, not determined using the multiplex PCR method.

### MLVA Typing on French Respiratory Specimens over Three Years in an Endemic Setting

Between October 1^st^ 2007 and September 30^th^ 2010, 35 patients were positive for *M. pneumoniae* using a specific real-time PCR on their respiratory tract specimens. Thirty-four specimens, including throat swabs, nasopharyngeal and bronchial aspirates, sputum, broncho-alveolar lavages and pleural fluids, corresponding to 30 patients, were available for MLVA typing (Table 2). *M. pneumoniae* MLVA profiles were obtained for 29 specimens, leading to a sensitivity of 85% for the MLVA typing method on clinical specimens. Fifteen distinct MLVA types were identified, 13 previously reported types (B, E, H, I, J, M, O, P, T, U, V, 27, and profile 24672 termed MLVA-29) [16], [17] as well as two new MLVA types, termed MLVA-30 for profile 34662 and MLVA-31 for profile 84472.

**Table 2:** Characteristics of 34 *M. pneumoniae*-positive respiratory tract specimens collected at the University Hospital of Bordeaux, France over three years (October 2007–September 2010) and of the corresponding *M. pneumoniae* isolates grown from them.

*M. pneumoniae*-positive clinical specimens											Corresponding *M. pneumoniae* isolates							
Specimen designation	Source^a^	Date of collection	Cycle threshold^b^	Mpn 1	Mpn 13	Mpn 14	Mpn 15	Mpn 16	MLVA type	Macrolide-resistance genotype^c^	Isolate designation	Mpn 1	Mpn 13	Mpn 14	Mpn 15	Mpn 16	MLVA type	Macrolide-resistance genotype
Mpn-3790	BAL	Oct. 07	36.7	4	3	6	6	2	O	wt	B4690	4	3	6	6	2	O	wt
Mpn-3817	Sputum	Oct. 07	20.9	8	4	4	7	2	31	wt	B4692	8	4	4	7	2	31	wt
Mpn-3823^e^	Throat	Nov. 07	21.3	3	4	5	7	2	J	wt	B4709	3	4	5	7	2	J	wt
Mpn-3825^e^	Pleural liq.	Nov. 07	29.2	3	4	5	7	2	J	wt	−d							
Mpn-3834	Sputum	Nov. 07	34.8	4	3	5	6	2	M	wt	–							
Mpn-3882	BAL	Jan. 08	25.4	2	3	5	6	2	B	wt	B4748	2	3	5	6	2	B	wt
Mpn-3884	Sputum	Jan. 08	31.1	2	3	5	6	2	B	wt	–							
Mpn-3920^f^	NPA	Jan. 08	34.0	2	3	5	6	2	B	wt	–							
Mpn-3909	Throat	Jan. 08	29.1	5	3	6	6	2	T	wt	B4774	5	3	6	6	2	T	wt
Mpn-3925	Throat	Feb. 08	35.9	3	3	6	6	2	H	wt	B4789	3	3	6	6	2	H	wt
Mpn-3969	Bronch. asp.	May 08	32.3	5	3	6	6	2	T	wt	B4811	5	3	6	6	2	T	wt
Mpn-4070	Throat	Jul. 08	37.1	6	3	5	6	2	V	wt	–							
Mpn-4074	BAL	Jul. 08	38.5	nd^g^	nd	nd	nd	nd	nd	no amp	–							
Mpn-4075	Bronch. asp.	Aug. 08	24.9	8	4	4	7	2	31	wt	B4879	8	4	4	7	2	31	wt
Mpn-4089^e^	Sputum	Sept. 08	25.3	4	3	6	6	2	O	wt	B4911	4	3	6	6	2	O	wt
Mpn-4097^e^	Throat	Sept. 08	35.2	4	3	6	6	2	O	wt	–							
Mpn-4087	Throat	Sept. 08	38.3	2	nd	nd	6	nd	nd	no amp	–							
Mpn-4139^f^	BAL	Dec. 08	34.9	5 h	4	5	7	2	U	A2059G	B4975	7	4	5	7	2	Z	A2059G
Mpn-4156	Bronch. asp.	Dec. 08	37.9	6	nd	5	6	2	nd	no amp	–							
Mpn-4161	Throat	Dec. 08	37.8	nd	nd	nd	nd	nd	nd	no amp	B4997	6	4	5	7	2	X	wt
Mpn-4194	BAL	Feb. 09	27.3	4	3	5	6	2	M	wt	B5029	4	3	5	6	2	M	wt
Mpn-4280	Throat	Apr. 09	22.6	2	4	5	7	2	E	wt	B5612	2	4	5	7	2	E	wt
Mpn-4279	Throat	Apr. 09	37.1	3	4	6	7	2	27	wt	–							
Mpn-4385	BAL	Jul. 09	23.1	4	4	5	7	2	P	wt	–							
Mpn-4392^e^	BAL	Aug. 09	24.5	2	4	6	7	2	29	wt	B5335	2	4	6	7	2	29	wt
Mpn-4393^e^	Bronch. asp.	Aug. 09	25.6	2	4	6	7	2	29	wt	B5336	2	4	6	7	2	29	wt
Mpn-4397	Throat	Aug. 09	36.1	3	4	6	6	2	30	wt	–							
Mpn-4445	Throat	Oct. 09	37.5	4	nd	5	nd	2	nd	no amp	B5475	4	3	5	6	2	M	wt
Mpn-4494	Throat	Dec. 09	37.0	3	3	6	7	2	I	wt	–							
Mpn-4524	NPA	Dec. 09	24.8	3	4	5	7	2	J	wt	B5596	3	4	5	7	2	J	wt
Mpn-4539	BAL	Jan. 10	26.9	2	4	5	7	2	E	wt	–							
Mpn-5493	Sputum	Feb. 10	26.9	2	3	5	6	2	B	wt	–							
Mpn-4693	Throat	Jul. 10	27.3	4	4	5	7	2	P	wt	B5674	4	4	5	7	2	P	wt
Mpn-4736	Throat	Sept. 10	33.0	5	4	5	7	2	U	wt	B5719	4	4	5	7	2	P	wt

a BAL, bronchoalveolar lavage; pleural liq., pleural liquid; NPA, nasopharyngeal aspirate; bronch. asp., bronchial aspirate.

b Cycle threshold from the in-house real-time PCR targeting the *M. pneumoniae* adhesin P1 used for primary detection of the pathogen [19].

c wt, wild type; no amp, no amplification with the real-time PCR used for the detection of 23S rRNA mutations associated with macrolide resistance according to Peuchant *et* *al.* [21]. Substitution A2059G (*E. coli* numbering) corresponds to substitution A2064G using *M. pneumoniae* numbering.

d No *M. pneumoniae* isolate grown from the specimen.

e Concurrent specimens from the same patient collected the same day.

f Sequential specimens from the same patient.

g nd, not determined.

h Inconsistencies concerning the marker Mpn1 leading to distinct MLVA types are underlined.

Three sets of two concurrent specimens collected from the same patient at the same time were analysed. In each case, MLVA typing led to an identical MLVA profile for both specimens (Table 2). It should be noted that the corresponding isolate grown from each of these specimens also harboured the same MLVA type (Table 2; specimens Mpn-3823/Mpn-3825, Mpn-4089/Mpn-4097 and Mpn-4392/Mpn-4393). One patient had two *M. pneumoniae*-positive specimens one year apart, in January and December 2008 (Table 2, specimens Mpn-3920 and Mpn-4139). MLVA typing distinguished MLVA type B and MLVA type U, respectively. Because those MLVA profiles differ by three distinct markers, it is plausible that this patient was re-infected by a different *M. pneumoniae* strain, which also harboured an A2059G substitution (see below).

An *M. pneumoniae* isolate could be grown from 19 respiratory tract specimens (culture sensitivity  = 56%). The MLVA profiles from specimens and isolates were concordant in all except two cases. In both cases (Table 2, specimen Mpn-4736/isolate B5719 and specimen Mpn-4139/isolate B4975), we noted a change in the number of tandem repeats of marker Mpn1, varying by two or one repeats, respectively, and leading to a change of the MLVA type. This discrepancy in the number of repeats at the Mpn1 locus was confirmed using the nested PCR method applied to these clinical specimens and *M. pneumoniae* isolates.

Macrolide resistance-associated mutations were searched for in all respiratory tract specimens. Twenty-nine (85%) genotypes were obtained. Twenty-eight (96.6%) specimens produced a melting peak characteristic of the wild-type genotype, and only one specimen (3.4%) harboured a macrolide-resistant A2059G genotype (*Escherichia coli* numbering, corresponding to A2064G using *M. pneumoniae* numbering) (Table 2, specimen Mpn-4139). This specimen was collected from a three-year-old boy recurrently hospitalised for acute exacerbation of asthma and pneumonia. From this specimen, corresponding macrolide-resistant isolate (isolate B4975) was obtained.

### MLVA Typing on Israeli Specimens Collected during a Surge of *M. pneumoniae* Respiratory Tract Infections

While for the French specimens a constant rate of isolation was observed during the 3 years of the study, a surge of *M. pneumoniae*-associated respiratory tract infections was observed in 2010 at the Hadassah-Hebrew University Medical Centre in Jerusalem. Fifty-five patients had a positive *M. pneumoniae* PCR in 2010, compared to 20 patients in 2007, two patients in 2008 and none in 2009 [6]. This finding led to the question of whether such epidemic surge was a monoclonal phenomenon. *M. pneumoniae* MLVA profile was obtained for 49 throat swab samples out of 63 (sensitivity of 78%), corresponding to 41 patients (Table 3). Interestingly, MLVA typing showed 18 distinct MLVA types, suggesting that the increased incidence of *M. pneumoniae* was a multiclonal phenomenon. The MLVA type O was the most frequent type (9 patients or 22%), followed by the MLVA type P (5 patients or 12%) and the MLVA type Z (4 patients or 10%). No association between the MLVA type and the month of isolation was noted. Four patients had a couple of concurrent or subsequent specimens that led to an identical MLVA type (Table 3, patients #4, #16, #26, #33). All five subsequent specimens that were obtained over 23 days in patient #2 were MLVA type Z, except one, which was categorised as MLVA type X. This change was due to a single tandem repeat difference for marker 1. Taking into account the possible instability of this marker reported above, we think that the most plausible hypothesis is that this patient was infected by a unique MLVA type Z *M. pneumoniae* isolate. However, we cannot exclude the possibility of a mixed infection by MLVA types Z and X *M. pneumoniae* in this patient.

**Table 3:** Characteristics of 49 *M. pneumoniae-*positive throat swab specimens collected at the Hadassah-Hebrew University Medical Center, Jerusalem, Israel in 2010, from 41 patients.

**Specimen designation**	**Patient number**	**Date of collection**	**Mpn 1**	**Mpn 13**	**Mpn 14**	**Mpn 15**	**Mpn 16**	**MLVA type**	**Macrolide-resistance genotype** a
P3001246	1	February 3	3	4	6	6	2	30	wt
P3001235^b^	2	February 1	7	4	5	7	2	Z	wt
P3001259^b^	2	February 8	6	4	5	7	2	X	wt
P3001279^b^	2	February 11	7	4	5	7	2	Z	A2058A/G^c^
P3001306^b^	2	February 22	7	4	5	7	2	Z	A2058G
P3001309^b^	2	February 23	7	4	5	7	2	Z	A2058G
P3001305	3	February 22	4	4	5	7	2	P	wt
P3001433^d^	4	March 18	2	3	6	6	2	C	wt
P3001434^d^	4	March 18	2	3	6	6	2	C	wt
P3001451	5	March 23	7	4	5	7	2	Z	A2058G
P3001482	6	April 6	3	3	6	6	2	H	wt
P3001487	7	April 6	5	3	6	6	2	T	wt
P3001502	8	April 11	4	4	5	7	2	P	wt
P3001531	9	April 15	4	3	6	6	2	O	wt
P3001538	10	April 18	4	4	5	7	2	P	wt
P3001561	11	April 25	5	3	6	6	2	T	wt
P3001600	12	May 3	7	4	5	7	2	Z	A2058G
P3001625	13	May 13	2	4	5	7	2	E	wt
P3001626	14	May 13	2	4	5	7	2	E	wt
P3001634	15	May 17	4	4	5	7	2	P	wt
P3001726^b^	16	June 8	4	3	6	6	2	O	wt
P3001741^b^	16	June 13	4	3	6	6	2	O	wt
P3001794	17	June 29	4	3	6	6	2	O	wt
P3001810	18	July 4	4	3	6	6	2	O	A2058A/G^c^
P3001823	19	July 7	7	4	5	7	2	Z	A2058G
P3001844	20	July 14	6	4	5	7	2	X	wt
P3001845	21	July 15	4	3	6	6	2	O	A2058G
P3001858	22	July 20	2	3	6	6	2	C	A2058G
P3001909	23	August 5	3	3	5	6	2	G	A2058G
P3001923	24	August 9	1	4	5	7	2	A	wt
P3001928	25	August 9	4	3	6	6	2	O	wt
P3001936^b^	26	August 11	5	3	5	6	2	S	wt
P3001940^b^	26	August 12	5	3	5	6	2	S	wt
P3001939	27	August 12	6	3	5	6	2	V	wt
P3002039	28	October 3	5	4	5	7	2	U	wt
P3002040	29	October 3	5	3	5	6	2	S	wt
P3002068	30	October 11	6	3	6	6	2	W	wt
P3002077	31	October 13	5	4	5	7	2	U	wt
P3002079	32	October 13	2	3	6	6	2	C	wt
P3002081^b^	33	October 17	4	3	6	6	2	O	wt
P3002263^b^	33	November 29	4	3	6	6	2	O	wt
P3002160	34	October 31	4	4	5	7	2	P	wt
P3002161	35	October 31	3	4	5	7	2	J	wt
P3002167	36	November 1	5	4	5	7	2	U	wt
P3002175	37	November 3	5	3	5	6	2	S	wt
P3002185	38	November 8	2	3	5	6	2	B	wt
P3002202	39	November 11	3	4	5	6	2	K	no amp
P3002234	40	November 22	4	3	6	6	2	O	wt
P3002291	41	December 7	4	3	6	6	2	O	A2058G

a wt, wild type; no amp, no amplification. Nucleotide A2058 (*E. coli* numbering) corresponds to nucleotide A2063 using *M. pneumoniae* numbering.

b Sequential specimens from the same patient.

c A2058A/G: simultaneous finding of both the macrolide-sensitive (2058A) and the macrolide-resistant (2058G) genotypes. (Dual peaks were found in raw data from the sequencing of domain V region of *M. pneumoniae* 23 S rRNA).

d Concurrent specimens from the same patient.

A macrolide resistance-associated mutation A2058G (*E. coli* numbering, corresponding to A2063G using *M. pneumoniae* numbering) was found in nine patients (22%). The comparison between the macrolide-resistant genotype and the MLVA type revealed that macrolide-resistant strains were found in four distinct MLVA types, type Z, O, C and G. MLVA type Z was the most frequent resistant type infecting four patients (44%). These patients, patients #2, #5, #12 and #19, were aged 6, 10, 31 and 6 years, respectively. In contrast to MLVA type O, in which 6 out of 9 patients had a wild-type genotype, in MLVA type Z, all 4 patients were shown to have an A2058G substitution in the 23 S rRNA gene. Notably, in patient #2 for whom the case report was previously published [5], the macrolide resistance emerged during a treatment with azithromycin.

## Discussion

*M. pneumoniae* is a fastidious bacterium that has a low detection sensitivity in culture. For this reason, a typing method that can be applied directly to patient specimens is of great need. MLVA typing methods are usually applied to bacterial isolates. In this study, the MLVA typing method of *M. pneumoniae* isolates was adapted by increase of sample amount and numbers of PCR cycles to be applicable directly to clinical specimens without the need for positive culture. With our method, the MLVA profile of *M. pneumoniae* could be obtained directly from respiratory tract specimens in 72–85% of cases. Specimens that failed to amplify had low levels of *M. pneumoniae* DNA. As a comparison, other *M. pneumoniae* PCR-based methods applied directly to clinical specimens, such as detection of macrolide resistance-associated mutations, also had a sensitivity below 80% [21], [22] and/or a required minimum bacterial DNA load [11].

Comparison of the results of MLVA typing between respiratory tract specimens and *M. pneumoniae* isolates grown from them led to two cases of discrepancy concerning the marker Mpn1. Moreover, the MLVA typing of five subsequent specimens from patient #2 of the Israeli cohort may also suggest a slight instability of marker Mpn1. The marker Mpn1 is 12 bp long and is located in the *hsdS* gene. It is the most discriminatory VNTR, with repeat copy numbers ranging from 1 to 8. This marker was previously shown to be stable by us [16] and others [17] in vitro after repeated culture passages or after infection passage in guinea pigs and in vivo using concurrent isolates. Moreover, other studies using the same marker Mpn1 for MLVA typing from specimens did not report any instability [2], [3], [17]., We suggest here that the number of tandem repeats of marker Mpn1 may vary by one or two repeats in a small number of cases. However, the possibility of mixed infection by different MLVA type strains could also explain these cases of discrepancy.

MLVA typing of *M. pneumoniae* is important both individually and epidemiologically. At the level of an individual patient, it allows the discrimination of relapse or persistence from repeated infections. In the first case, the MLVA type remains identical. In case of a re-infection, the MLVA type is likely to be different. At the population level, MLVA typing assesses the diversity of *M. pneumoniae* strains that are circulating in a community or during an epidemic surge. In this study, we showed that the *M. pneumoniae* spread was polyclonal in Bordeaux between 2007 and 2010, without any predominating MLVA type. This observation could be attributed to the absence of an epidemic surge of *M. pneumoniae* observed there during this period. This diversity of *M. pneumoniae* MLVA types was previously reported in France [16]. The respiratory tract specimens from Israel were collected during an epidemic surge of *M. pneumoniae* infections. However, in contrast to the clonal spread that could have been expected, the MLVA typing revealed that this epidemic surge was a multi-clonal phenomenon, with 18 distinct circulating MLVA types. Three MLVA types, O, P and Z, were predominant and were found in 44% of patients. MLVA types O and P were previously shown to be frequent MLVA types [16]. On the contrary, MLVA type Z is not a frequent MLVA type because it was found before in only 1.9% of *M. pneumoniae* isolates [16]. Interestingly, macrolide resistance was found or was generated during the course of infection in all the patients infected with an MLVA type Z strain. The patients were not related since they came from different families, neighbourhoods and religious communities. Although the number of those patients remained limited, there might be an association between the MLVA type Z and macrolide resistance in Jerusalem. However, it is not possible to conclude whether MLVA type Z strains are more prone to acquire macrolide resistance-associated mutations or whether a macrolide-resistant *M. pneumoniae* clone is spreading in Jerusalem. One study reported a strong association between the P1 gene PCR-RFLP type 1 and erythromycin resistance in Shanghai [23]. However, because the macrolide-resistance rate is high in Asia [18] and because the PCR-RFLP typing method only separates two different types, these resistant strains may not necessarily belong to the same clone. In Beijing, type 1 macrolide-resistant *M. pneumoniae* isolates were recently shown to belong to different clones based on the study of a single VNTR that exists in P1 cytadhesin gene (MPN141) [24]. Finally, the determination of both the macrolide-resistant genotype and the MLVA type needs to be continued on *M. pneumoniae*-positive respiratory tract specimens collected in Jerusalem to assess the possibility of an association between the MLVA type Z and macrolide resistance.

In summary, the discriminatory power of the MLVA method showed that the spread of *M. pneumoniae* strains in France over three years in an endemic setting was polyclonal. Moreover, the surge of *M. pneumoniae* infections in Israel in 2010 was not attributed to the spread of a single clone but was also a polyclonal phenomenon.
